# Nurse Documentation of Child Weight-Related Health Promotion at Age Four in Sweden

**DOI:** 10.3390/nursrep11010008

**Published:** 2021-02-02

**Authors:** Anna Svensson Sehic, Mikaela Persson, Eva K. Clausson, Eva-Lena Einberg

**Affiliations:** Faculty of Health Sciences, Kristianstad University, S-291 88 Kristianstad, Sweden; anna78_s@hotmail.com (A.S.S.); mikaelajohansson6@hotmail.com (M.P.); eva.clausson@hkr.se (E.K.C.)

**Keywords:** Child Health Service, documentation, health-promotive interventions, overweight, obesity, record review

## Abstract

(1) Background: Overweight and obesity in children have increased worldwide and tend to persist into adolescence and adulthood. The Child Health Service (CHS) has an important role in providing health-promotive interventions, and such interventions are required to be documented in a child’s health record. The aim of the study was to investigate Child Health Care (CHC) nurses’ documentation of weight-related, health-promotive interventions in the Child Health Care Record (CHCR) regarding lifestyle habits in connection to the four-year visit. (2) Methods: A record review of 485 CHCRs using a review template was accomplished. Of the included CHS units, four used electronic records and two used paper records. Chi-square tests and Spearman’s rank-order correlations were used to analyse data. (3) Results: The results showed that CHC nurses document interventions regarding lifestyle habits to a low extent, although children with overweight/obesity seemed to undergo more interventions. There was also a difference between electronic and paper records. (4) Conclusions: The consequences of not documenting the interventions in the CHCR make it difficult to follow up and demonstrate the quality of the CHC nurse’s work. There is a need for more research to gain a deeper understanding of the reasons that the work of CHC nurses is not visible in children’s health records.

## 1. Introduction

The prevalence of overweight and obesity in children has increased dramatically, and these conditions tend to persist into adolescence and adulthood [1,2,3]. Overweight and obesity increase the risk of health-related lifestyle diseases, such as adult metabolic syndrome, diabetes and stroke [4,5]. In 2019, 38 million children under the age of 5 were overweight or obese [3]. In Sweden, approximately 11% of four-year-olds [6] and 21% of six- to nine-year-olds [7] are overweight or obese. Children’s lifestyle habits have changed in terms of diet and physical activity, which has contributed to an increased proportion of children with overweight and obesity [8,9]. An unhealthy lifestyle with overweight and obesity can lead to other physical consequences, such as asthma, dental problems and metabolic risk factors later in life [5,10]. A child’s lifestyle habits are influenced by the family’s food and activity habits, which are a fundamental part of the child’s health development [9]. Natural play in children tends to be a low priority because of increased screen time, which is currently strongly linked to obesity in younger children. Previous studies showed that newborn children are exposed to regular screen time, and 90% of children under two years of age watch TV daily. Extensive use of media may adversely affect children’s sleep, and getting fewer than 12 h of sleep per night can be associated with higher body mass index (BMI) among three-year-old children [11]. Since a child’s lifestyle habits are established at an early age, it is important to focus on health-promotive interventions early on.

The Child Health Service (CHS) has a mission to promote children’s health and development and to prevent ill health through monitoring and health guidance, such as providing information, advice and support regarding lifestyle habits to all children, regardless of disease history or other special needs [12,13]. In health promotion work, Child Health Care (CHC) nurses provide interventions to children based on child and family needs. During each child’s four-year visit, CHC nurses are responsible for initiating a health dialogue about the family’s lifestyle habits, such as diet, physical activity, sleep and media usage. CHC nurses also monitor the child’s growth and BMI [12,13]. The level of the health-promotive interventions given by CHC nurses is based on the child’s BMI and parents’ knowledge about lifestyle factors [14]. Each enrolled child in the CHS has an individual health record, the Child Health Care Record (CHCR), which is mainly important for the child but is also used for follow-up and quality assurance of care [13]. Due to a shift in the CHS record system in Sweden, the CHCR consists of paper records or electronic records, and its efficacy is questioned because the record systems lack a clear overview and good quality for monitoring [15]. Previous studies showed a shortage of documentation in CHCR regarding the child’s health, defined as incomplete and unclear records. The health-promotive interventions performed by CHC nurses may not be visible in the CHCR due to ethical aspects, e.g., from a psychosocial perspective or family function, and lack of time and/or lack of knowledge regarding documentation [16]. Even in recent studies, shortcomings in documentation emerged [17]. When health-promotive interventions regarding lifestyle habits are not documented or performed to the extent the child and their family demands, the child’s health may suffer [18]. Since there is a trend of increasing unhealthy habits, there is a need to examine which health-promotive interventions are documented in the CHCR to determine whether children and their families receive the information, advice and support that they deserve. Therefore, the aim of the study was to investigate CHC nurses’ documentation of weight-related, health-promotive interventions in the CHCR for four-year-old children regarding lifestyle habits such as diet, physical activity, sleep and TV/media usage.

## 2. Methods

### 2.1. Design and Settings

The study was conducted as a retrospective, cross-sectional study [19] in both rural and urban areas in the southern part of Sweden. The region has approximately 1.5 million inhabitants, of which there are 18,500 registered four-year-old children (Statistics Sweden, 2019). In Sweden, CHS reaches almost 100% of all children aged 0–5 years old [13]. The study complies with STROBE reporting guidelines for observational studies.

### 2.2. Data Collection

Information letters about the study were sent to the head of 25 health centres with CHS. Oral information was given by phone one week after the information was sent, and written consent was retrieved by six CHS units, of which four used an electronic CHCR and two used a paper CHCR. Targeted information to parents about the study was displayed visually in the waiting room at the CHS unit. To gain access to the electronic CHCR, assistance from CHC nurses was required at the respective CHS unit. The access was limited to the CHCR system and information about the current year cohorts for the study.

The CHCRs of children born in 2012 and 2013 were included (*n* = 485). The inclusion criteria were the presence of a CHCR in which it was clearly documented that a four-year visit took place. In Sweden, the four-year visit consists of a health dialogue, monitoring the child’s growth, development, hearing and vision, as well as a follow-up of the child’s and family’s situation. CHCRs indicating that the four-year visit was not completed for some reason but resumed on another occasion were included if it was clearly apparent that the appointment was related to the requisite four-year visit.

#### Template for Review

The review template is based on the CHC programme for four-year-old children [13]. The main content of the template concerns the child’s BMI, parents’ height and weight, preschool participation, status and interventions about diet, physical activity, sleep, TV/media usage, parents’ lifestyle habits and other healthcare providers. Preschool participation, parents’ lifestyle habits and other healthcare providers were not included in this study. The review was based on data collection with tick-off, meaning a dash was noted in the review template for each found documentation subjected for the review; a review template per record was used. A pilot study was conducted to test the quality of the review template. The pilot study examined ten electronic records. The template for review met the criteria for answering the aim of the study and was therefore not changed. The included CHCRs were reviewed jointly and equally by two of the authors (M.P. and A.S.S.) to minimize any risks, such as misinterpretations.

The following questions guided the review:

1. Does the CHC nurse’s documentation regarding diet, physical activity, sleep and TV/media habits differ depending on whether the documentation is performed in electronic records or in paper records?
2. Do the CHC nurse’s documented health-promotive interventions differ based on the child’s BMI?

### 2.3. Statistical Analyses

Data were analysed using descriptive statistics with frequencies (n) and percentages (%) to describe the results. Chi-square tests were used to investigate differences between status regarding lifestyle habits, health-promotive interventions and record system, as well as the child’s BMI. Spearman’s rank-order correlations were used to investigate correlations between documented interventions and the record system and correlations between documented interventions and the child’s BMI. For correlation coefficients, the value 1.00 stood for perfect positive correlation, 0 stood for no correlation and −1.00 for perfect negative correlation. Values close to 1.00 or, on the other side of the range, close to −1.00 could be interpreted as strong correlations and values close to 0 were interpreted as weak correlations (19). The BMI classifications were designed according to established limit values for underweight, normal weight, overweight and obesity of the respective sex at age four [12]. When few records included BMI classification obesity, the overweight and obese subgroups were merged into a new subgroup. In 19 records (3.9%), calculated BMI was missing. These records were excluded from the analyses where the child’s BMI was a variable. The significance level was set to *p* < 0.05. Statistical analysis was performed using IBM SPSS version 24 (IBM, Armonk, NY, USA).

### 2.4. Ethical Considerations

Ethical guidelines by the World Medical Association Declaration of Helsinki were followed. The collected data were anonymized and strictly locked in without access for unauthorized persons. The Regional Ethics Committee in Lund, Sweden, approved the study (case no. 2014/200).

## 3. Results

Of 485 reviewed CHCRs, 31% were paper records and 69% were electronic records. In only three CHCRs, there was information about both the height and weight of the child’s parent. Either the height or the weight of the parents was documented in nearly 30% of the records. In 87.3% of the paper records, there was a calculated BMI. In 100% of the electronic records, there was a calculated BMI, as it is calculated automatically in electronic records. The frequencies of documented variables regarding the children and their parents in paper and electronic records are presented in Table 1.

Documentation of status regarding dietary habits occurred in almost 90% of all CHCRs, while interventions regarding diet occurred in nearly 25% of the CHCRs. The status of physical activity was documented in 74% of all CHCRs, while interventions on physical activity were found in 16.7% of the CHCRs. Documentation of status regarding sleep habits occurred in nearly 65% of all CHCRs. Of all lifestyle habit areas in both record systems, sleep habits were the area with the least documented interventions (13.8%). Documentation regarding TV/media usage status was found in fewer than 50% of all CHCRs, while interventions on TV/media usage were found in 15% of all CHCRs. The status of dietary habits was documented to a greater extent in electronic records, while documented interventions were more commonly found in paper records. The electronic records had over 35% more documentation about the status of physical activity than the paper records. Significant differences between documentation in paper records and electronic records were found regarding the status of diet, physical activity and TV/media habits and for interventions about diet and sleep. Documentation regarding status and interventions comparing between paper and electronic records is demonstrated in Table 2.

In 414 CHCRs (88.9%), it was documented that the children had normal weight. Overweight was documented in 35 (7.5%) CHCRs, and obesity was documented in 17 CHCRs (3.6%). In the CHCRs wherein normal weight was documented, there were documented diet interventions in 22% of the records; in contrast, in the CHCRs wherein overweight/obesity was documented, documentation about diet intervention was presented in 61.5% of the records. The record review showed that 32.7% of the children with overweight/obesity received physical activity interventions compared with the children with normal weight who received less than half as many interventions (15.2%). The record review also showed that CHCR where overweight/obesity were documented contained fewer documented data on status and interventions regarding sleep compared with CHCR where normal weight was documented. Interventions regarding TV/media were documented in 5 CHCR of a total of 52 where BMI classification overweight/obesity was documented. Significant differences were observed between CHCR where normal weight was documented and CHCR where overweight/obesity was documented regarding diet and physical activity interventions. No significant differences were observed for the other variables. Documentation regarding status and interventions in relation to the child’s BMI classification is demonstrated in Table 3.

The results of Spearman’s rank-order correlation indicated that there was a low negative correlation between documented BMI classification and documented interventions regarding diet and physical activity (rs = −0.287, *p* = <0.001; rs = −0.157, *p* = 0.001, n = 466, respectively). Children with normal weight do not receive interventions regarding diet and physical activity to the same extent as children with overweight or obesity. Spearman’s rank-order correlation indicated that there was a low positive correlation between the recording system and interventions regarding diet and sleep (rs = 0.136, *p* = 0.003; rs = 0.094, *p* = 0.038, n = 485, respectively). Paper records more often contain documentation regarding diet and sleep interventions in comparison with electronic records.

## 4. Discussion

The results showed a low frequency of documentation of health-promotive interventions provided to four-year-old children and their families. Only every fourth child was given diet interventions according to what was documented. In terms of physical activity, sleep and TV/media, there were even fewer documented interventions. The reason that diet interventions occurred more often than other types of intervention may be that diet is considered to have a direct link to overweight and obesity. This suggests that CHC nurses prioritize providing more interventions related to diet than those related to physical activity, sleep and TV/media usage. To support CHC nurses in providing interventions in all aspects of lifestyle habits, a national health dialogue model was developed, namely, “Establish Healthy Habits” [12,20]. Studies show that interventions may not be provided when necessary because CHC nurses feel that overweight and obesity in children is a sensitive topic to discuss [18,21,22]. The results in the present study, i.e., the low number of documented interventions, may be explained by the fact that CHC nurses feel uncomfortable talking about lifestyle habits. A previous study showed that a good, trustworthy relationship between CHC nurses and family was important and facilitated CHC nurses’ work to provide health-promotive interventions [23]. Previous studies also found that CHS nurses lack the time and knowledge to engage in health-promotive work with children and parents [21,22]. This could be another explanation as to why the results indicate that CHC nurses document few interventions. The importance of pedagogical tools was highlighted previously [20,24]. The “Establish Healthy Habits” guidelines [12,20], a pedagogical tool for engaging in health dialogues with children and their families, is currently available for CHC nurses. However, we do not know the extent to which it is used. In addition to the availability of guidelines and tools, nurses also need supervision and opportunity for skills training to feel confidence in health-promotive work [20,24]. Sjunnestrand et al. [22] argued that CHC nurses need continuing education, supervision and training in communication skills, as well as more time to talk with families with children who are overweight or obese. Future studies investigating CHC nurses’ documentation of health-promotive interventions may find different results regarding the development of national guidance, which has occurred over recent years within the CHS.

In all lifestyle habit areas, interventions were more often documented in paper records. Documentation regarding status occurred more frequently in the electronic records. The reason for this difference between the record systems is not stated in the results; however, the record system may be significant and have an effect on CHC nurses’ health-promoting work. A child’s BMI is automatically calculated in the electronic record and therefore also documented in 100% of cases; in contrast, in the paper record, BMI was missing in nearly 17% of the records. Ståhl [16] stated that the record system is of importance and that electronic records are more reliable because they accurately reflect the needs of the child; this suggestion did not quite correspond to the results of the present study, wherein interventions were more often documented in paper records. The results showed that there are few documented interventions regardless of record system, but it is unclear whether CHC nurses provide more information verbally than what is documented in the record. Previous research showed that CHC nurses provide more information verbally in encounters with children and their families than what is documented in the record [16]. Differences between what is communicated and what is documented were found to be in both directions, i.e., what is communicated is not documented, and what is documented is not communicated [25]. The Patient Data Act [26] prescribes that CHC nurses have an obligation to document, and what is not documented did not occur. Studies showed that the documentation problem is also found among school nurses who find that the structure of the record, lack of time and tradition limit the ability to document [27]. Ståhl [16] agreed that lack of time and the structure of the record limits the possibility of documenting a coherent overall picture of the child. A more accurate documentation that gives an overall picture of the child’s situation and given measures enables a reliable follow-up and transfer of information to other healthcare providers, such as school health services. Given the importance of documentation for both the child and CHS, there is a need to improve routines and knowledge about documentation. The development of electronic records may facilitate the documentation of given health-promotive interventions. However, there is a risk of less detailed documentation when using a highly structured journal where the majority of the documentation is done by choosing between different possible answers instead of documenting by writing in free text. This could also lead to limitations in the documentation and omission of the resulting information. To avoid this, it is important that CHC nurses actively participate in the development of how the CHCR should be structured to facilitate the documentation of, for example, interventions.

According to the record review of CHC nurses’ documentation, overweight and obese children receive more interventions in terms of diet and physical activity than children with normal weight. Sleep and TV/media interventions were given less often based on what is documented, regardless of the child’s BMI classification. All children, irrespective of BMI, should receive health-promotive interventions in all lifestyle areas. On the other hand, the children with the greatest need for interventions are given priority in the field of child welfare [13]. Based on this, children with overweight/obesity should be prioritized, which was not found in the CHC nurses’ documentation, where only one in ten overweight/obese children received interventions related to sleep and TV/media. In a previous study, the CHC nurses experienced that the parents’ lack of interest was a barrier in communication that affected the possibility of providing interventions to overweight children [23]. Therefore, the attitude of the parents could be an aspect that affects the CHC nurses’ health-promotive work. Another study found that parents were more susceptible to counselling and support in certain areas of life. For example, parents were more likely to make dietary changes than changing TV/media habits [28]. Throughout the present study, a clear trend showed that CHC nurses document fewer interventions about sleep and TV/media than diet and physical activity, which is remarkable, as there is research that shows a clear association between sleep deprivation and childhood obesity [29]. Due to the important interactions between physical activity, sedentary behaviour and sleep time, the World Health Organization [30] developed guidelines regarding this for children under five years of age. If the recommendations are followed, it is expected to promote children’s development and lifelong health. Asante et al. [28] suggested that parents today request more support and advice from the CHS regarding healthy lifestyle choices for the whole family. This indicates that regardless of whether the child has normal weight or overweight/obesity, parents place higher demands on the CHS to respond to each individual family’s needs. We know that the CHS reaches almost all children in Sweden and that childhood overweight/obesity has increased dramatically. Therefore, CHC nurses need to focus more on health-promotive interventions related to lifestyle habits.

A total of 25 CHS units were asked to participate in the study, of which only six chose to participate. Most of the CHS units did not return despite repeated attempted contact. Possible reasons for this may be a lack of time, a holiday period and changes in managers. Furthermore, the personnel experienced that the study was preventing their work, they needed to assist with access to CHCR and provide room for the authors. It is also conceivable that nurses felt hesitant to expose the enrolled children. The experience was that managers protected CHC nurses who, in turn, protected children and parents; these phenomena are referred to as gatekeeping [31]. Gatekeeping in healthcare is common, especially when it involves smaller children [32]. It could also be discussed if the CHC nurses felt examined in their work. To encourage CHC nurses to participate in similar studies in the future, the purpose of the study and how it could benefit CHS as well as the work of CHC nurses need to be further clarified.

The nonvalidated review template worked well and can be considered credible since it is based on the Children’s Health Care Program for four-year-olds [12]. It is estimated that if the study were carried out a later date, the same results would be obtained. However, to enrich the results of this study and acquire knowledge of what is documented in free text regarding interventions, a content analysis could have been made as a supplement. A limitation of this study is that we do not know if there are any differences due to the documentation between the CHS units that participated and those who did not. Furthermore, this study did not include any information on the variability of the documentation between nurses. Some nurses may have documented at higher rates and others at lower rates. Generalization of these results is therefore not possible. However, the results can serve as a basis for further studies and can be used for the development and quality improvement of CHC nurses’ documentation regarding health-promotive interventions. CHC nurses’ work may then also be more visible.

## 5. Conclusions

The results showed that CHC nurses documented few health-promotive lifestyle interventions in connection to four-year visits. There is a need to improve the documentation of CHC nurses’ health-promoting work so that interventions for children and families appear in the CHCR. Since almost 100% of all children participate in four-year visits, CHS has a unique opportunity to influence the future health of both children and families. This is especially important in view of the increasing problems of overweight and obesity at an early age. Failure to document the interventions makes it difficult to follow up and ensure the quality of CHC nurses’ work in CHS. More studies are considered necessary in the field to gain a deeper understanding of the aspects that make the work of CHC nurses invisible in CHCR. From a legal perspective, what is not documented did not occur.

## Figures and Tables

**Table 1:** Frequencies of documented variables regarding the child and their parents.

	Paper Record N* = 150	Digital Record N* = 335	Total N* = 485
Calculated BMI for the child	131 (87.3)	335 (100)	466 (96.1)
Parents’ height and weight,	2 (1.3)	1 (0.3)	3 (0.6)
Mother’s height or weight	52 (34.7)	92 (27.5)	144 (29.7)
Father’s height or weight	50 (33.3)	90 (26.9)	140 (28.9)

*N = number of records (%).

**Table 2:** Documentation of status and interventions regarding lifestyle habits in relation to record system.

	Paper Record N* = 150	Digital Record N* = 335	Total N* = 485	*p*-Value **
Diet status	119 (79.3)	310 (92.5)	429 (88.5)	**<0.001**
Diet interventions	53 (35.3)	75 (22.4)	128 (26.4)	**0.003**
Physical activity status	73 (48.7)	286 (85.4)	359 (74)	**<0.001**
Physical activity interventions	30 (20)	51 (15.2)	81 (16.7)	0.192
Sleep status	97 (64.7)	218 (65.1)	315 (64.9)	0.931
Sleep interventions	28 (18.7)	39 (11.6)	67 (13.8)	**0.038**
TV/media status	24 (16)	182 (54.3)	206 (42.5)	**<0.001**
TV/media interventions	24 (16)	49 (14.6)	73 (15.1)	0.696

*N = number of records (%); ** Chi-square test, *p* < 0.05 were considered significant and marked in boldface.

**Table 3:** Documentation of status and interventions regarding lifestyle habits in relation to the child’s BMI.

	Normal Weight N* = 414	Overweight/Obesity N* = 52	Total N* = 466	*p*-Value **
Diet status	367 (88.6)	45 (86.5)	412 (88.4)	0.654
Diet interventions	91 (22)	32 (61.5)	123 (26.4)	**<0.001**
Physical activity status	307 (74.2)	42 (80.8)	349 (74.9)	0.300
Physical activity interventions	63 (15.2)	17 (32.7)	80 (17.2)	**0.002**
Sleep status	272 (65.7)	30 (57.7)	302 (64.8)	0.254
Sleep interventions	59 (14.3)	6 (11.5)	65 (13.9)	0.595
TV/media status	182 (44)	24 (46.2)	206 (44.2)	0.764
TV/media interventions	68 (16.4)	5 (9.6)	73 (15.7)	0.203

*N = number of records (%); ** Chi-square test, *p* <0.05 were considered significant and marked in boldface.

## References

[1] Geserick M., Vogel M., Gausche R., Lipek T., Spielau U., Keller E., Pfäffle R., Kiess W., Körner A. (2018). Acceleration of BMI in early childhood and risk of sustained obesity. N. Engl. J. Med..

[2] NCD-Risk Factor Collaboration [NCD-RisC] (2017). Worldwide trends in body-mass index, underweight, overweight, and obesity from 1975 to 2016: A pooled analysis of 2416 population-based measurement studies in 128.9 million children, adolescents, and adults. Lancet.

[3] Obesity and Overweight. https://www.who.int/news-room/fact-sheets/detail/obesity-and-overweight.

[4] Kim J., Lee I., Lim S. (2017). Overweight or obesity in children aged 0 to 6 and the risk od adult metabolic syndrome: A systematic review and meta-analysis. J. Clin. Nurs..

[5] Reilly J.J., Kelly J. (2011). Long-term impact of overweight and obesity in childhood and adolescence on morbidity and premature mortality in adulthood: Systematic review. Int. J. Obes..

[6] Spong E., Miregård J., Nylander C. Övervikt och Fetma Bland 4-Åringar i Sverige [Overweight and Obesity Among 4-Year-Olds in Sweden]. https://samverkan.regionsormland.se/for-vardgivare/halsoval/barnhalsovard/rapporter-fran-barnhalsovarden-sormland/.

[7] Övervikt och Fetma är Vanligt och ökar Med Åldern hos 6–9 Åringar [Overweight and Obesity is Common and Increases with Age of 6-9-Year-Olds]. https://www.folkhalsomyndigheten.se/contentassets/4b83de69772549f7ac477979bebd55af/20100-overvikt-fetma-6-9-aringar.pdf.

[8] Moraeus L., Lissner L., Olsson L., Sjöberg A. (2015). Age and time effects on children’s lifestyle and overweight in Sweden. BMC Public Health.

[9] Stenhammar C. (2011). Parental Perspectives on Preschool Children´s Lifestyle—Quantitative and Qualitative Aspects. Ph.D. Thesis.

[10] Pulgarón E.R. (2013). Childhood Obesity: A Review of Increased Risk for Physical and Psychological Comorbidities. Clin. Ther..

[11] Dattilo A.M., Birch L., Krebs N.F., Lake A., Taveras E.M., Saavedra J.M. (2012). Need for Early Interventions in the Prevention of Pediatric Overweight: A Review and Upcoming Directions. J. Obes..

[12] Rikshandboken i Barnhälsovård [Digital National Handbook for Child Health Services]. https://www.rikshandboken-bhv.se/.

[13] Swedish National Board of Health and Welfare Vägledning för Barnhälsovården [Guideline for Child Healthcare]. https://www.socialstyrelsen.se/globalassets/sharepoint-dokument/artikelkatalog/vagledning/2014-4-5.pdf.

[14] Huus K. (2009). Weight Gain in Children—Possible Relation to the Development of Diabetes. Ph.D. Thesis.

[15] Magnusson M., Lindfors A., Tell J. (2011). Stora skillnader i svensk barnhälsovård [Large differences in Swedish Child Health care]. Läkartidningen.

[16] Ståhl Y. (2012). Documentation in Child and School Health Services. Mapping Health Information from a Biopsychosocial Perspective Using the ICF-CY. Ph.D. Thesis.

[17] Köhler M., Rosvall M., Emmelin M. (2016). “All is well”: Professionals’ documentation of social determinants of health in Swedish Child Health Services health records concerning maltreated children—A mixed method approach. BMC Pediatrics.

[18] Regber S. (2014). Barriers and Facilitators of Health Promotion and Obesity Prevention in Early Childhood: A Focus on Parents. Ph.D. Thesis.

[19] Polit D.F., Beck C.T. (2017). Nursing Research. Generating and Assessing Evidence for Nursing Practice.

[20] Håkansson L. (2015). Sjuksköterskans Erfarenhet av Samtal om Hälsosamma Matvanor inom Barnhälsovården [The Nurse’s Experience of Conversations about Healthy Eating Habits in Child Health Care]. Master’s Thesis.

[21] Isma G.E., Bramhagen A.-C., Ahlström G., Östman M., Dykes A.-K. (2013). Obstacles to the prevention of overweight and obesity in the context of child health care in Sweden. BMC Fam. Pract..

[22] Sjunnestrand M., Nordin K., Eli K., Nowicka P., Ek A. (2019). Planting a seed—Child health care nurses’ perceptions of speaking to parents about overweight and obesity: A qualitative study within the STOP project. BMC Public Health..

[23] Edvardsson K., Edvardsson D., Hörnsten Å. (2009). Raising issuses about childrens overweight. J. Adv. Nurs..

[24] Golsäter M., Enskär K., Lingfors H., Sidenvall B. (2009). Health counselling: Parental-oriented health dialogue—An innovation for child health nurses. J. Child Health Care.

[25] Turer C.B., Barlow S.E., Montaño S., Flores G. (2017). Discrepancies in communication versus documentation of weight-management benchmarks: Analysis of recorded visits with latino children and associated health-record documentation. Glob. Pedaitric Health.

[26] (2008). The Patient Data Act (SFS 2008:355).

[27] Clausson E.K., Berg A., Janlöv A.-C. (2015). Challenges of documenting schoolchildren’s psychosocial health—A qualitative study. J. Sch. Nurs..

[28] Asante P.A., Cox J., Sonneville K., Samuels R.C., Taveras E.M. (2009). Overweight Prevention in Pediatric Primary Care: A Needs Assessment of an Urban Racial/Etnic Minority Population. Clin. Pediatrics.

[29] Fatima Y., Doi S.A.R., Mamun A.A. (2015). Longitudinal impact of sleep on overweight and obesity in children and adolescents: A systematic review and bias-adjusted meta-analysis. Obes. Rev..

[30] Guidelines on Physical Activity, Sedentary Behaviour and Sleep for Children under 5 Years of Age. https://apps.who.int/iris/bitstream/handle/10665/325147/WHO-NMH-PND-2019.4-eng.pdf?sequence=1&isAllowed=y.

[31] Williams P. (2020). ‘It all sounds very interesting, but we’re just too busy!’: Exploring why ‘gatekeepers’ decline access to potential research participants with learning disabilities. Eur. J. Spec. Needs Educ..

[32] Coyne I. (2010). Accessing children as research participants: Examining the role of gatekeepers. Child Care Health Dev..

